# A series of unfortunate events: Long‐neglected malpositioned pacemaker and its consequences

**DOI:** 10.1002/ccr3.7776

**Published:** 2023-08-07

**Authors:** Abdelrahman Osman, Ali Ahmad

**Affiliations:** ^1^ University of Cincinnati College of Medicine Cincinnati Ohio USA; ^2^ Mercy Health Tiffin Hospital Tiffin Ohio USA

**Keywords:** cardiology, cardiothoracic surgery, infectious diseases, surgery

## Abstract

Use of pacemakers has increased in recent years, and they have become the premier treatment option for several arrhythmias. However, they are not without risk; this case report aims to show the importance of follow‐up and adherence to guidelines, and methods to diagnose a life‐threatening complication.

## Graphical abstract

Four‐chamber view TEE showing pacemaker ventricular lead crossing the atrial septum and going down through the mitral valve (left). Modified 3‐chamber view showing ventricular lead going through mitral valve and inserting into lateral wall of left ventricle (right).

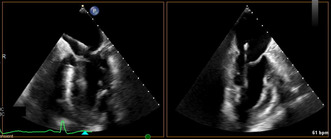

## 1 INTRODUCTION

Cardiac device therapy, including pacemakers and defibrillators, is an integral part of the treatment of bradyarrhythmia, tachyarrhythmia, and advanced heart failure. An aging population, increasing prevalence of cardiovascular disease, advances in device therapy, and introduction of implantable heart rate‐rhythm monitoring devices have resulted in an exponential gross in therapeutic cardiac device implantation worldwide. More than 300,000 pacemakers are inserted every year in the United States alone.^1^ These devices improve the quality of life and increase survival in many cases. However, device implantation is not without risk, and long‐term monitoring of patients with device therapy is crucial to achieve benefit and to avoid potential immediate and long‐term complications.

Despite the multiple clinical scenarios in which permanent pacing is considered, most management decisions regarding permanent pacemaker implantation are driven by the association of symptoms with bradycardia. Other common indications include sick sinus syndrome (SSS), high‐grade atrioventricular block, and tachycardia‐bradycardia syndrome. It is not uncommon that pacemaker therapy is considered for the treatment of chronotropic incompetence, but it is rarely considered in patients with cardio‐inhibitory neurocardiogenic dysfunction.^2^

Permanent transvenous pacing system leads are guided through the left subclavian, cephalic, or axillary veins and eventually screwed into place in the right ventricle.^3^ After placement, cardiac follow‐up is necessary to rule out inadvertent lead placement, thromboembolic events, or other possible medical emergencies.

We are presenting an extremely rare case of a 53‐year‐old man with an old, neglected, and misplaced right ventricular (RV) pacemaker lead, caused by a series of unfortunate events involving lack of follow‐up and neglect of the recommended guidelines before, during, and after device implantation.

## 2 CASE REPORT

A 53‐year‐old man with a history of coronary artery disease, hypertension, hyperlipidemia, and SSS presented to the emergency room for evaluation of dizziness and diaphoresis.

The patient had a history of pacemaker implantation at 26 years old after a motor vehicle accident presumably secondary to dizziness and SSS. After implantation, the patient was unable to follow up with a cardiologist or primary care provider for 27 years. He claims this is because he works as a truck driver and never settled in one place. He has no insurance and usually seeks medical advice only when he has an acute illness. He never established care with a primary care provider or a cardiologist.

**FIGURE 1:**
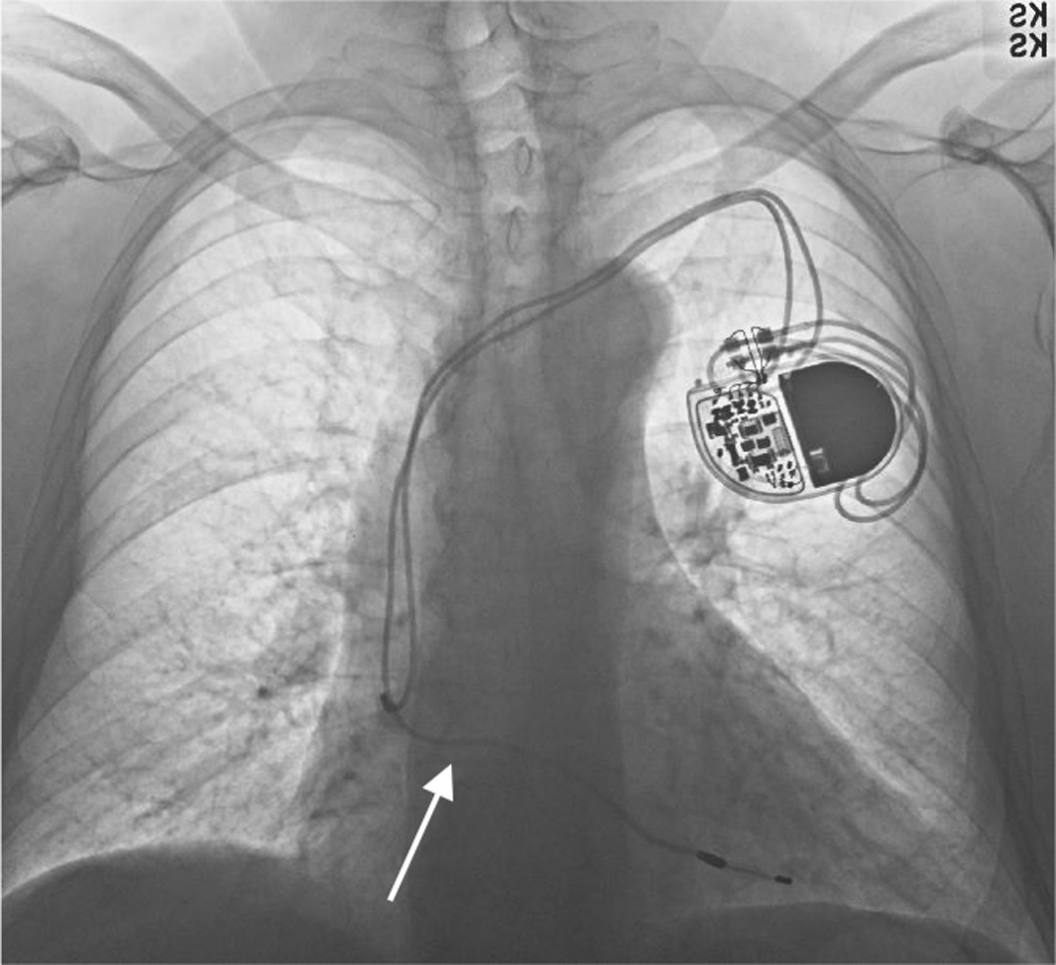
AP chest X‐ray showing pacemaker lead taking a sharp, straight turn (arrow) through the septum before resting in the left ventricle of the heart.

**FIGURE 2:**
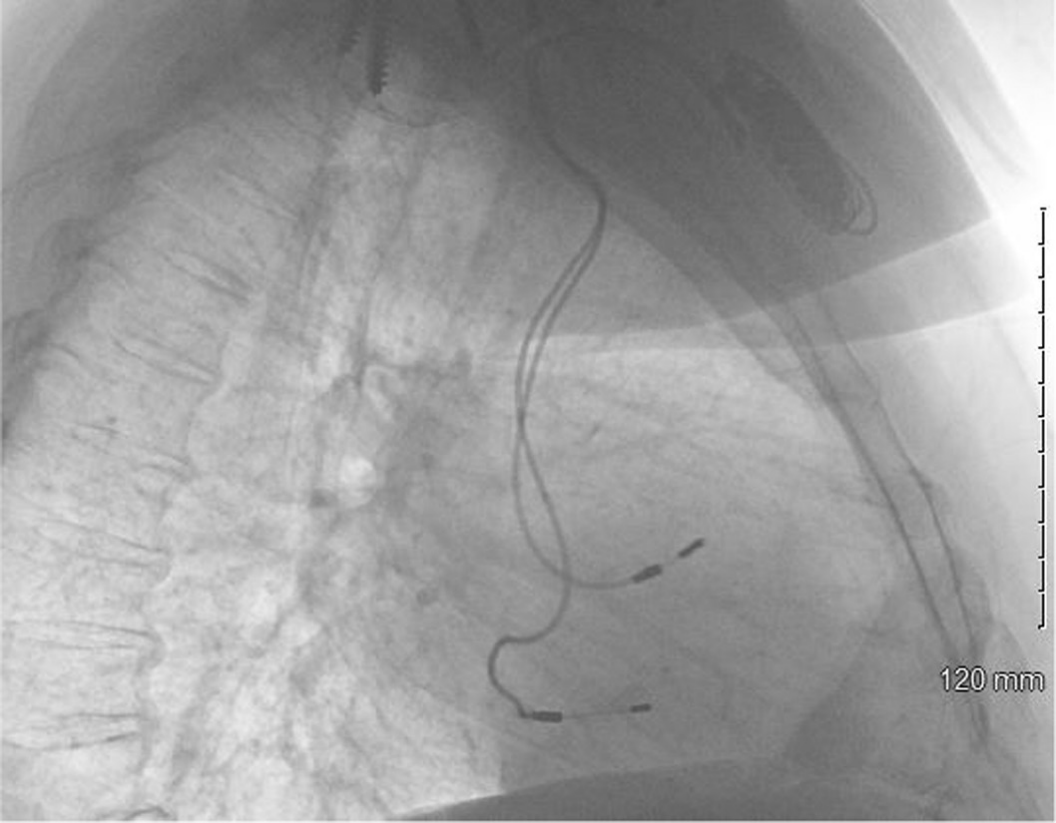
Lateral chest X‐ray showing pacemaker lead tips projected anteriorly, in a seemingly correct position.

In the emergency room, a point‐of‐care ultrasound revealed normal ejection fraction and wall motion, as well as sinus bradycardia at rest. No images of the ultrasound were recorded, and thus, no comment on the position of the pacemaker leads was made. An AP and lateral chest X‐ray were also obtained; despite showing the ventricular lead taking a sharp turn and crossing into the left chamber of the heart in the AP view (Figure 1), the lateral view was deceiving and showed the lead in a normal position (Figure 2). No comment of the lead position was made on either radiograph.

**FIGURE 3:**
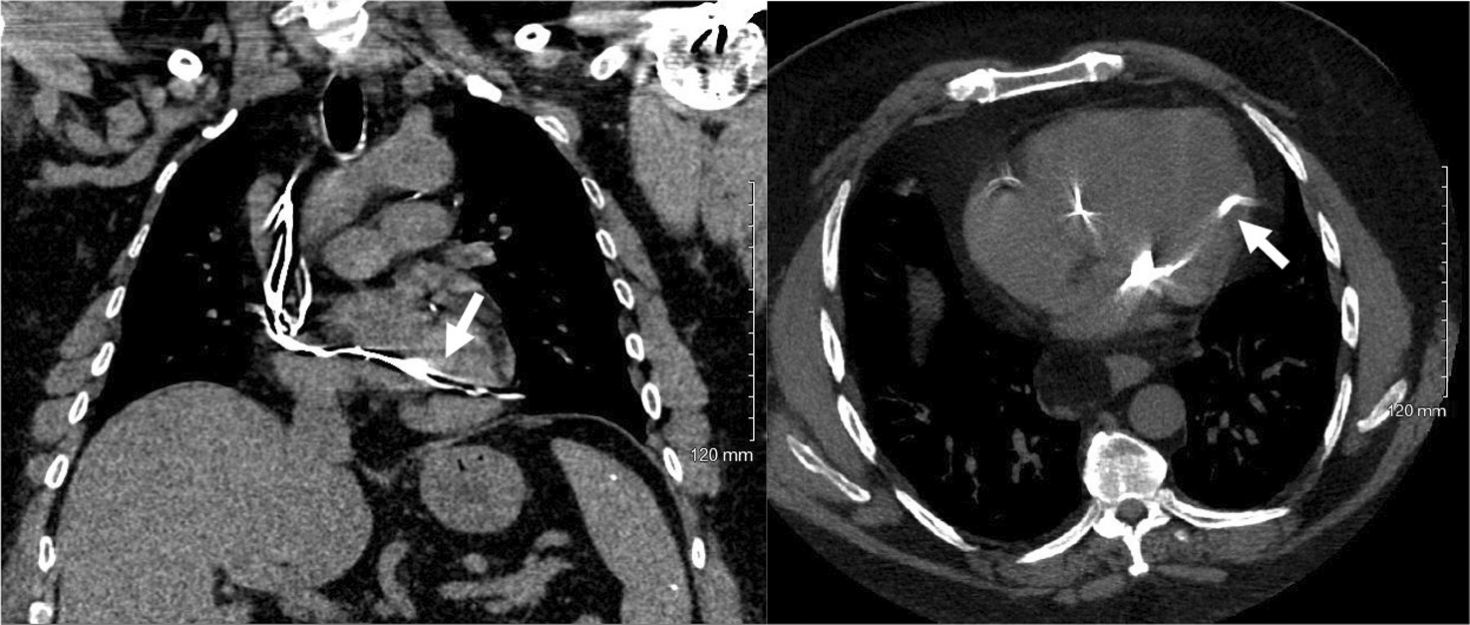
Chest CT without contrast in the coronal (left) and axial (right) plane. Despite no remark on it, the pacemaker lead is seen crossing into the left ventricle (arrows).

A chest CT without contrast clearly showed the RV lead crossing the interatrial septum, crossing the mitral valve, and finally inserting into the left ventricle (Figure 3). Again, these findings were missed by the reading radiologist because the indication of the study was to rule out pneumonia. Thus, no comment on the misplaced ventricular pacemaker lead was stated.

Because of his dizziness and resting sinus bradycardia, pacemaker interrogation was attempted, but unfortunately was unsuccessful due to dead pacemaker battery. Because of his significant orthostatic dizziness and persistent sinus bradycardia in the low 50s bpm at rest, along with suboptimal heart rate response in mild‐to‐moderate activities, we discussed replacing the patient's pacemaker battery. No formal exercise stress test was done to document chronotropic incompetence.

Patient underwent a pacemaker generator change and his baseline pacing rate was set at 70 bpm. He was atrial paced most of the time and, per intra‐operative testing, both atrial and ventricular leads were electrically intact. Unfortunately, neither the operator nor the pacemaker representative noted that the pattern of the ventricular pacing lead during testing had a right bundle branch block (RBBB) pattern.

**FIGURE 4:**
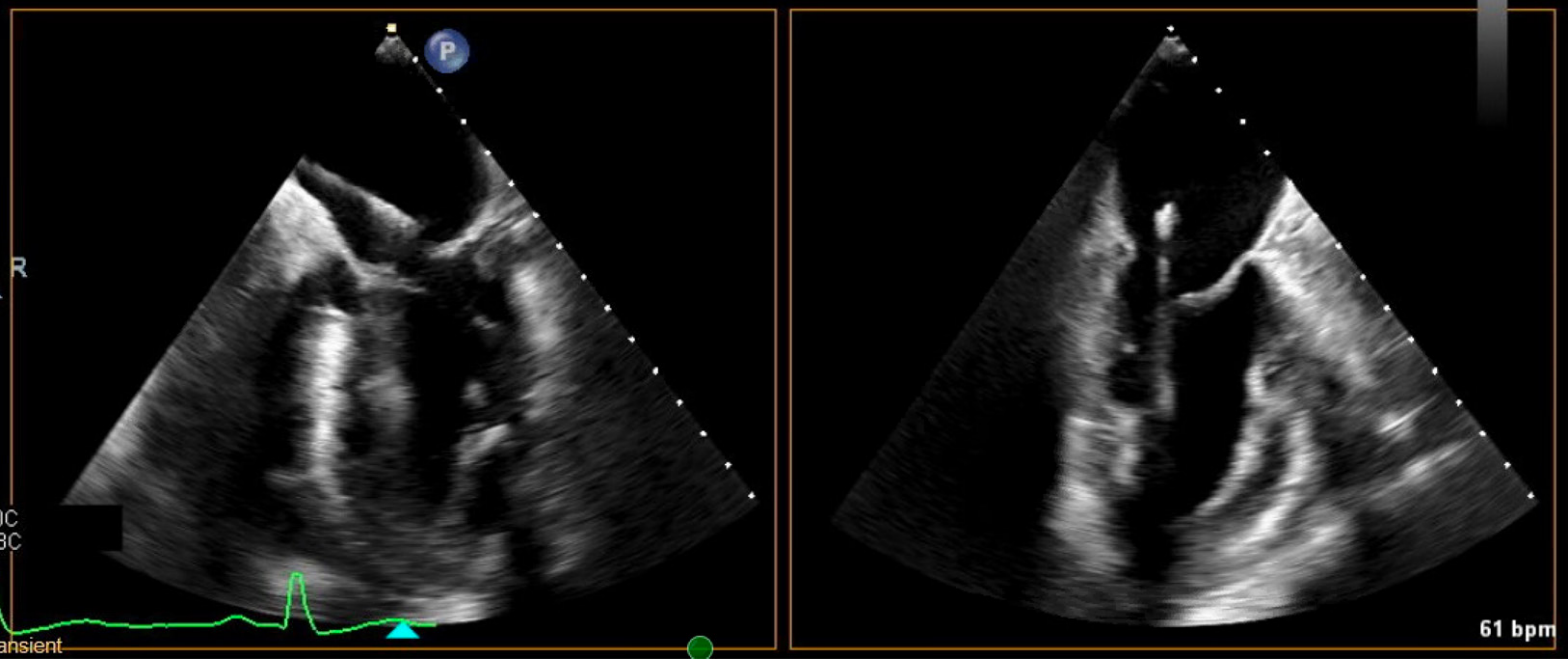
Four‐chamber view TEE showing pacemaker ventricular lead crossing the atrial septum and going down through the mitral valve (left). Modified three‐chamber view showing ventricular lead going through mitral valve and inserting into lateral wall of left ventricle (right).

**FIGURE 5:**
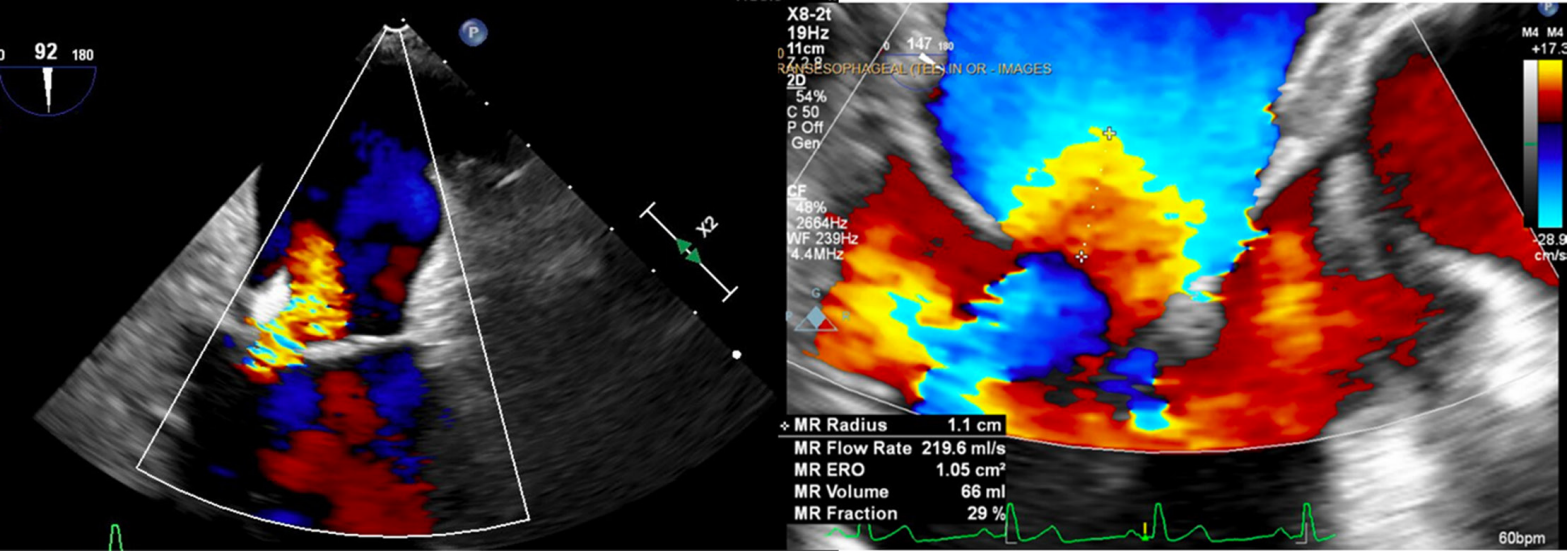
Zoomed view of mitral valve in two‐chamber view showing at least moderate mitral regurgitation caused by pacemaker lead interfering with closure of the mitral leaflets.

**FIGURE 6:**
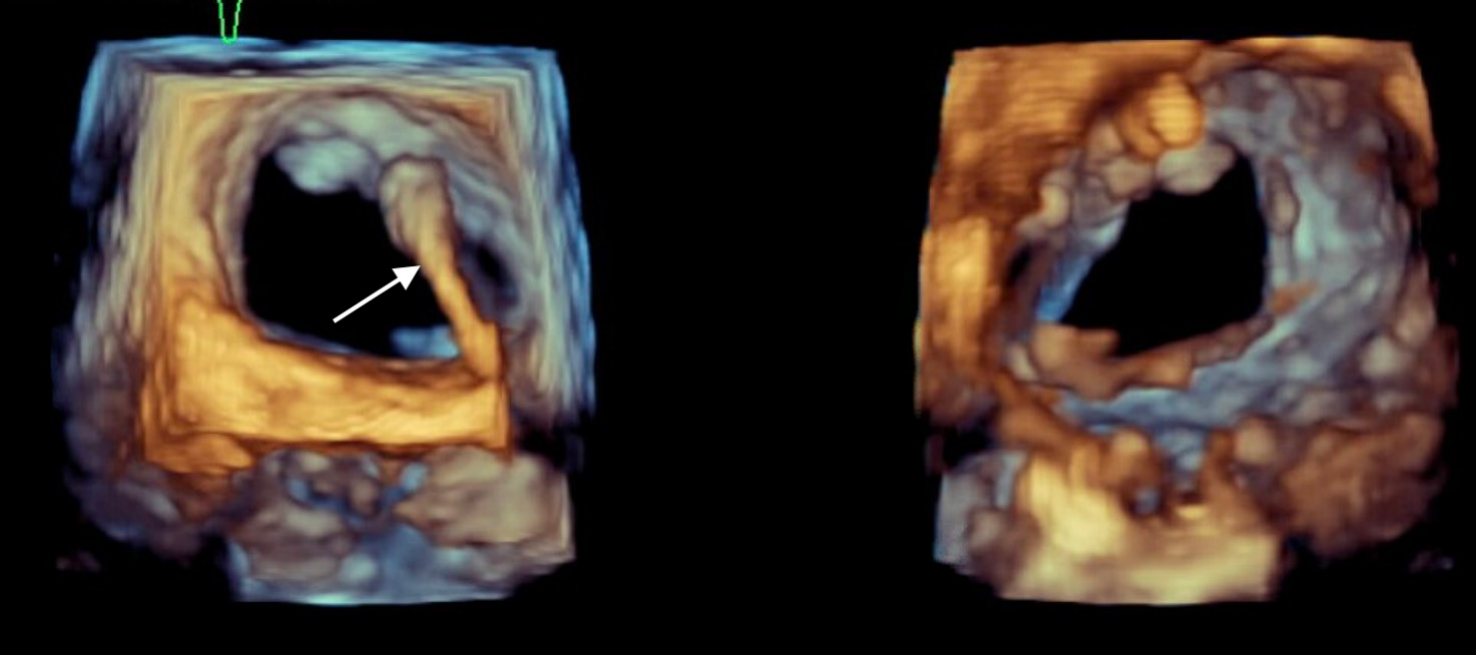
Three‐dimensional view zoom mode shows the ventricular lead (arrow) crossing through mitral valve from atrial face view (left) and ventricular face view (right).

A week later, the pacemaker pocket was found to be infected with Klebsiella organism. The decision was made to remove the pacemaker generator. A transesophageal echo (TEE) was subsequently performed in preparation for removal of the long‐neglected leads. Imaging showed the ventricular pacemaker lead crossing through a patent foramen ovale into the left atrium, through the mitral valve, and inserting into the lateral wall of the left ventricle (Figure 4). The TEE detected no vegetation but showed the ventricular lead interfering with coaptation of the mitral valve leading to at least moderate mitral valve regurgitation (Figures 5 and 6). Once the decision was made for surgical extraction, the patient underwent coronary angiography to rule out coronary artery disease and was found to have 80% mid‐LAD stenosis.

**FIGURE 7:**
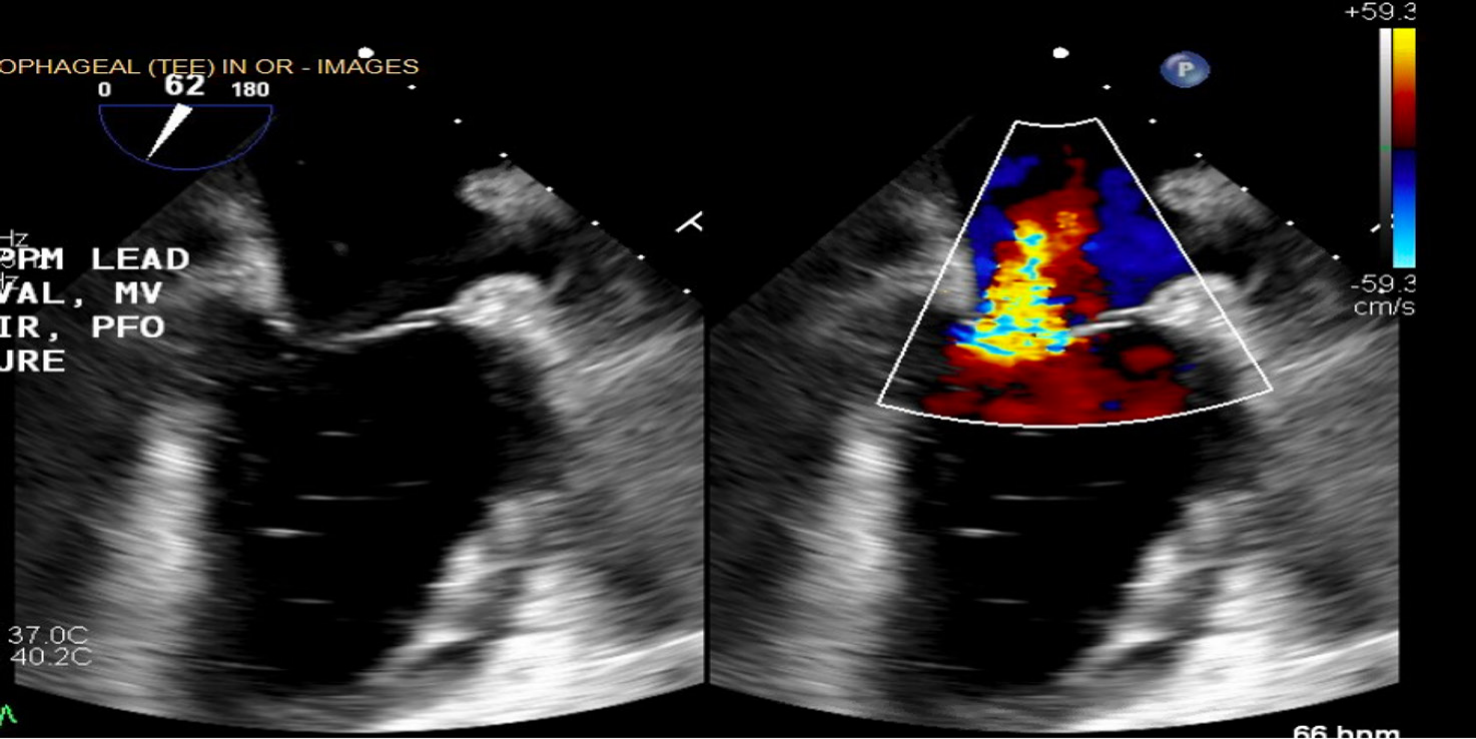
After extraction of pacemaker lead, persistent mitral valve regurgitation led to decision to repair mitral valve via closure of scallop.

**FIGURE 8:**
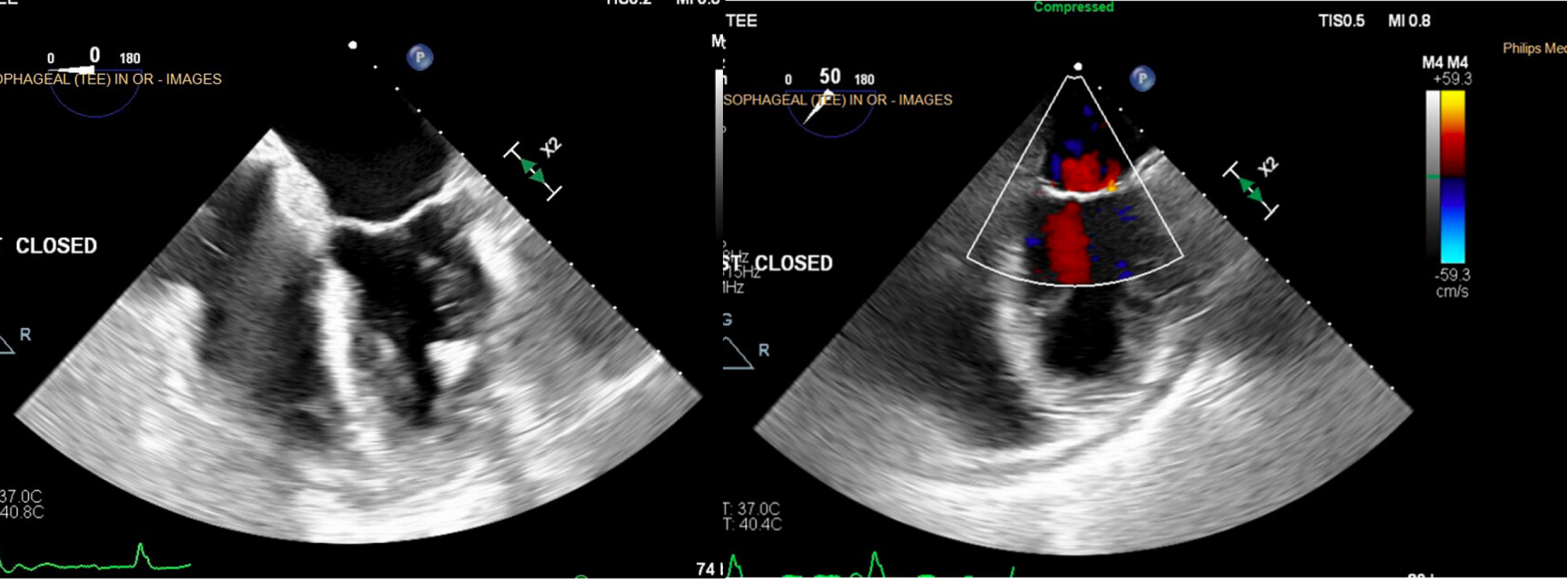
Post‐operative imaging revealed successful repair of the mitral valve and reduced mitral valve regurgitation.

A sternotomy was performed. The ventricular lead was inserted into the lateral wall of the left ventricle, while the atrial lead was properly positioned in the right atrium. Both leads were surgically removed. Despite extraction, mitral valve regurgitation was persistent (Figure 7). The mitral valve was thus repaired via closure of scallop via Alfieri stitch lead. In addition, the patent foramen ovale was closed. Patient also had CABGx1 with LIMA to LAD for an 80% stenosis of the mid LAD. Post‐operative imaging showed successful extraction of leads and repair of the mitral valve (Figure 8).

After pacemaker extraction, an EP study was performed to determine eligibility for another pacemaker insertion. However, studies showed normal sinus node function (1050 ms, CSNRT 200 ms), normal AV node function (AH 95 ms), and mildly prolonged HV interval (65 ms). There is no evidence of dual AV node physiology or accessory pathway. VA conduction was present. The decision was thus made to not implant another pacemaker. The patient is in general good health post‐operatively with no further dizziness or lightheadedness, and no chronotropic incompetence.

## 3 DISCUSSION

Pacemaker or ICD implantation is one of the most common cardiac interventions utilized today. They are used to treat cardiac arrythmia, including bradycardia and tachycardia. As such, indications for pacemaker implantation are numerous. ICD and pacemakers are most used to treat sinus node dysfunction (SND) and atrioventricular block. They can also treat chronic bifascicular block, hypertrophic cardiomyopathy, or patients with congenital heart disease.^2^ The need for a pacemaker can easily be determined using ECG or EP studies. However, as manifested by this case, a high degree of skepticism is required, including situations with young patients or those who have few cardiac studies.

Pacemaker implantation is a minimally invasive procedure, but still carries risks. Nearly a third of all complications are due to lead dislodgement or malposition.^3^ A proper ventricular lead is one placed in the RV cavity; however, leads can migrate to the left ventricle (LV) through several pathways. Passage through an atrial septal defect is most common but can also occur through a patent foramen ovale or a ventricular septal defect.^4^ An RV lead in the LV is a serious and likely under‐reported complication of pacemaker implantation. Leads in the LV can lead to dangerous thromboembolic events, which can occur anywhere from months to years after lead migration.^3^

**FIGURE 9:**
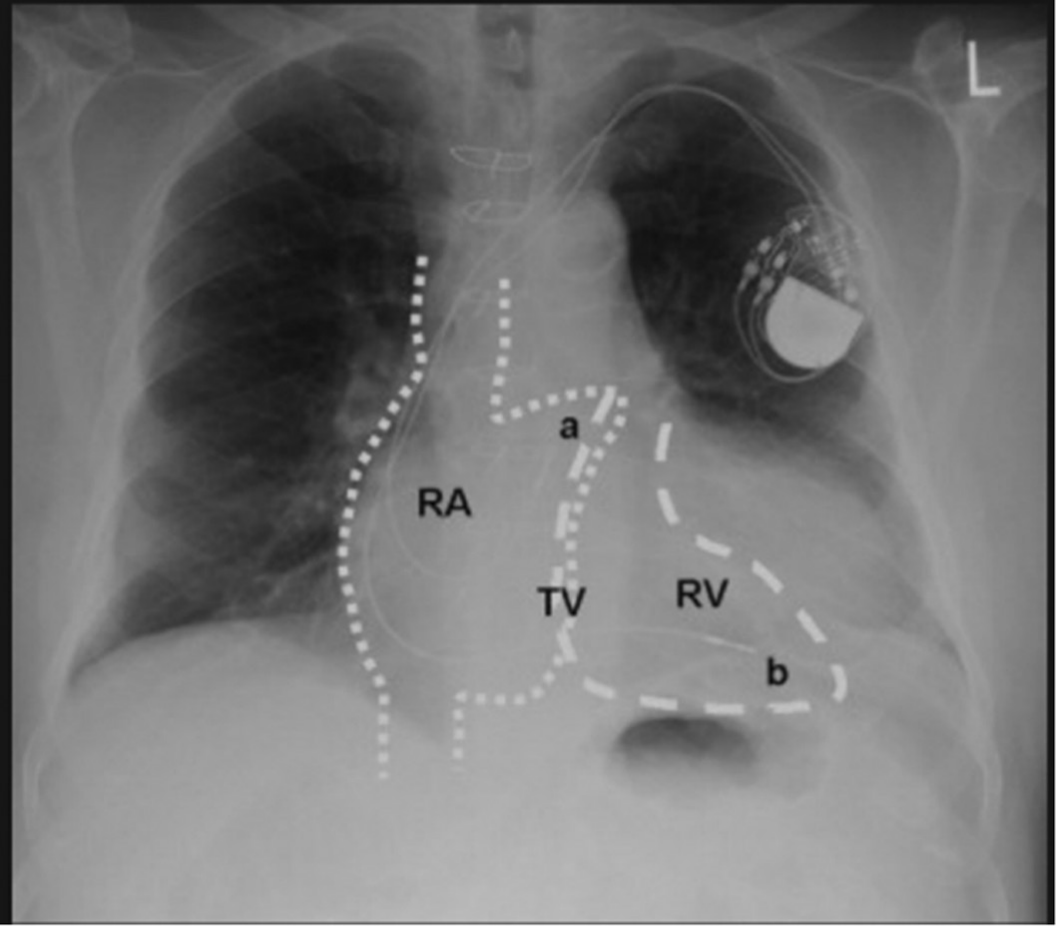
AP chest X‐ray with representation of chamber anatomy.^7^ The ventricular lead (b) is seen coursing along the right lateral aspect of the heart, before eventually bowing at the apex and lodging in the RV. The atrial lead (a) is projected over the right atrium (RA).

**FIGURE 10:**
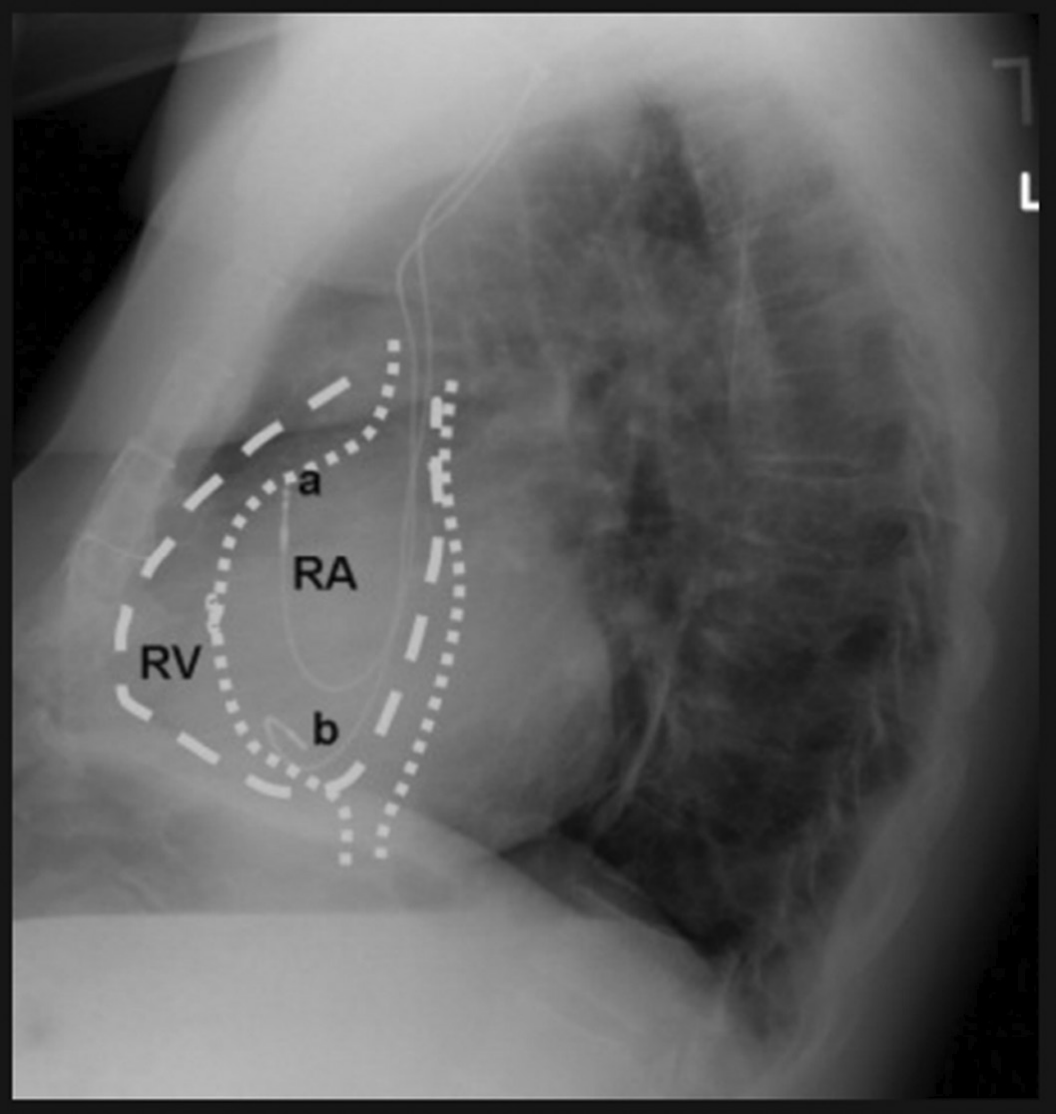
Lateral chest X‐ray with representation of chamber anatomy.^7^ The ventricular lead (b) and atrial lead (a) are both seen projecting anteriorly.

A misplaced ventricular lead must quickly be diagnosed to prevent adverse events, and thus, a high degree of scrutiny is required. The most important tool to recognize a lead in the LV is an ECG; on ventricular pacing, a misplaced lead will display a RBBB morphology rather than the expected left bundle branch block (LBBB).^5^ However, this method is limited in cases such as SND in the absence of AV node disease, as the patient would likely only have atrial pacing or no pacing at baseline.^3^ Adjunctive imaging is used to further confirm a misplaced lead, with AP and lateral X‐rays as the primary techniques. A correctly positioned RV lead on an AP chest X‐ray should have a smooth right lateral course with slight bowing at the RV apex (Figure 9). A lateral chest X‐ray should display the tip of an ICD lead projected anteriorly (Figure 10); in a mispositioned LV lead, this tip is projected posteriorly.^6^ It may be difficult to differentiate between a lead in the LV from those in the middle cardiac vein or coronary sinuses. Thus, CT imaging or TEE can be used to visualize lead migration.

Management of a misplaced LV lead depends on symptoms and risks of intervention. Lead removal presents a risk of thromboembolic events, and so surgical removal is usually withheld from asymptomatic patients or patients in poor health. Anticoagulation therapy is currently the most popular method of treatment. However, if anticoagulation therapy is ineffective and thromboembolic complications occur, surgical intervention must be considered.^8^ In our case, surgical extraction was needed due to infected pacemaker pocket and because of the mitral regurgitation that was caused by the misplaced ventricular lead.

## 4 CONCLUSION

Pacemaker and ICD implantation is a useful therapy for many arrythmias, but proper review of all indications is required. Additionally, consistent follow‐up and comments on positioning of the lead is necessary, as inadvertent placement of a pacemaker or ICD into the left ventricle is a rare but under‐reported complication that carries a high risk of thromboembolic events and must be diagnosed and treated quickly. In this case, not all events should be attributed solely to patient non‐compliance. There is no clear documentation regarding why the pacemaker was indicated, and the malpositioned left ventricular lead should have been recognized early, likely before the patient was discharged from the hospital after implantation. There are various methods useful in recognizing such a complication, including ECG, AP and lateral chest X‐rays, TEEs, or CT scans.

## AUTHOR CONTRIBUTIONS

**Abdelrahman Osman:** Writing – original draft. **Ali Ahmad:** Conceptualization; data curation; formal analysis.

## FUNDING INFORMATION

No funding source supported this study.

## CONFLICT OF INTEREST STATEMENT

The authors declare no conflicts of interest.

## CONSENT STATEMENT

Written informed consent was obtained from the patient to publish this report in accordance with the journal's patient consent policy.

## Data Availability

Data sharing is not applicable as no new datasets were generated or analyzed.
